# Breaking Down Barriers: Findings from a Literature Review on Housing for People with Disabilities in Latin America

**DOI:** 10.3390/ijerph20064972

**Published:** 2023-03-11

**Authors:** Claudia Valderrama-Ulloa, Ximena Ferrada, Felipe Herrera

**Affiliations:** Centro de Investigación en Tecnologías para la Sociedad, Facultad de Ingeniería, Universidad del Desarrollo, Las Condes, Santiago 7610658, Chile

**Keywords:** co-words analysis, residential building, disabled people, physical disability, visual disability, intellectual disability

## Abstract

Accessibility to housing is crucial for people with disabilities as it provides them with equal opportunities and allows them to live independently. A systematic literature review has been conducted to understand the current research on accessibility in housing for people with disabilities in Latin America. The study analysed 56 papers and used co-word analysis to identify common themes and topics within the documents. The results of the analysis showed that Brazil (61%) is the country with the most research on the subject, physical disability, at 36%, is the impairment most analysed, and interventions or analysis for the older people (45%) in their homes is the most researched type of population. The co-word analysis revealed that topics such as policy, regulations, the use of technologies, ergonomics interventions, and architectural criteria or barriers to the daily life of disabled people were frequently discussed in the papers. Although this work shows a substantial and growing increase in research on housing for people with disabilities in Latin America, it also demonstrates the importance of increasing research on other types of impairment, such as visual and cognitive-intellectual disabilities, and including children, caregivers, or even young adults.

## 1. Introduction

The number of people with disabilities in the world has been increasing. Currently, 15% of the world’s population lives with a disability (more than 1000 million people) [1], and around 85 million people live in Latin America. This number is expected to continue to increase due to population growth and medical advances, extending the life and ageing process [2]. In addition, the ageing process of the population will require increasingly complex devices to face potential physical illness, disability, and high dependency rates in their daily lives.

The International Classification of Functioning (ICF) defines disability as deficiencies in body functions and structures, limitations in activities, or restrictions in participation [3]. Thus, disability will result from the interaction between bodily deficiencies or health conditions and personal, environmental, and internal factors. Because of that, housing and its architectural barriers can make a person with disabilities “disabled” or not.

Housing is considered a basic need and a human right that various international institutions have recognised (Declaration of Human Rights (Article 25 (1)) [4], the International Covenant on Economic, Social and Cultural Rights (Article 11) [5], and the Charter of Fundamental Rights of the European Union (Article 34 (3)) [6]. Regarding this, the 2030 agenda for sustainable development establishes that disability cannot be a criterion for demonstrating compliance regarding human rights [7].

Poor access to housing produces social exclusion, health risks, or lack of access to essential services. In contrast, poor housing quality is related to health, education, child development, and the general well-being of the population [8], especially for older adults or people with disabilities.

In general, most housing studies focus on older people [9,10,11,12] since this relationship is critical to keep the independence of the older people [13,14,15]. These studies analyse, for example, how to avoid the risks of falls [16,17,18], home adaptations for the older people [19,20], or how housing helps to sustain clinical support [21,22], leaving aside young adult and children with illnesses or disabilities who also require assessments and adaptations for their homes [23].

Different studies can be found on housing improvements for different disabilities, such as a study by Ahmad et al. [24] on home modifications for people in wheelchairs in Pakistan, the work of Brunnstrom et al. [25] regarding home lighting adaptations for people with vision problems in Sweden, or an international review of environmental interventions for people with Alzheimer’s, where no LATAM country was included [26]. Regrettably, there need to be more similar studies in Latin America. 

Also, even though the built environment and its effects on health have been widely studied [27,28,29,30], there has been little attention paid to accessible home environments for people with functional limitations [31].

Accessible housing can be conceived in the design or can be achieved later through modifications, allowing older persons with functional limitations, people with disabilities, as well as their caregivers and visitors to benefit from it.

On the other hand, there is an insufficient supply of housing in the world that meets accessibility standards, in addition to a lack of access to the means to purchase or rent accessible housing. However, do we know what accessible housing is? This information seems insufficient at the time of studying an offer and identifying, on the part of the user, if the housing is accessible. Therefore, many people with disabilities reside in housing that is not suitable for their needs, highlighting those people who acquired their disability over time and already resided in housing without an accessible design [32]. Thus, it is observed that in developed countries such as, for example, the United Kingdom, 70% of people with muscle-wasting conditions live in housing that is not adapted to their mobility needs and lack sufficient adaptations to use the bathroom or kitchen [33]; so, too, in Australia, with approximately 120,000 people participating in the National Disability Insurance Scheme indicating that they will not be able to reside in accessible housing [34]. Moreover, in the United States, a Harvard University report [35] highlights that there is an insufficient supply of rental housing units that are accessible, and, in most cases, residential housing has limited modifications to ensure universal accessibility.

Thus, accessible housing development and retrofit decisions require the coordination of various stakeholders, including builders, design and construction professionals, occupational therapists, families and caregivers of people with disabilities, and governmental and non-governmental health and social support housing providers. All the named groups should work together to reach a consensus on housing modification decisions and new developments, ultimately based on the needs and preferences of the end user.

The limited supply and insufficiency of accessible housing appear to be due to various factors, including public and private financial constraints, individual socioeconomic constraints, and a lack of coordination between the public and private sectors [36]. In part, the scarce supply of housing is a symptom of the complexity involved in the development of accessible housing units and the decisions to modify them, particularly due to (i) the slow implementation of new policies on accessibility or the multiple construction standards that projects must meet; (ii) the lack of accessibility, especially focused on older buildings or dwellings. It is indicated that those countries that even have laws on accessibility that are 20–40 years old confirm a low level of compliance; (iii) the lack of consultation with, and participation of, people with these needs; or (iv) the lack of rigorous and comparable data on disability [2].

The demand for accessible housing in LATAM will increase in the coming years, primarily due to (i) the increasing prevalence of functional limitations in an ageing population (by 2030, this population will represent 17% and, by 2050, 25% [1]); and (ii) this region having about 85 million people with disabilities (one in every three dwellings) [2]. It is, thus, interesting to assess how Latin American countries have responded to this challenge and in which areas there is room for improvement.

To accomplish this, the following paper performs a systematic review through the PRISMA Preferred Reporting Items for Systematic Reviews and Meta-Analyses methodology [37]. From this systematic review, we will seek to answer the question: which countries in LATAM are researching accessibility in housing, and for what type of disability?

Subsequently, based on a co-word analysis, we will identify which topics are being researched in LATAM.

## 2. Methods

This review was carried out according to the PRISMA 2009 checklist and methodology [37]. Although PRISMA is in high demand in medical journals, its checklist and flowchart allowed the authors to sequentially organise the tasks to be performed for this systematic review. Thus, the general concepts and topics covered by PRISMA are relevant for any systematic review, not only for those aimed at summarizing the benefits and harms of a health intervention [37]. PRISMA has shown that the quality of reports and methodologies improved significantly in many journals [38] and facilitated the readability and traceability of information [39]. Another benefit of its use is an improvement in the details of study designs, results, and measurements [40]. It has been seen that when a study mentions that they used PRISMA in their methodology, the completeness of the information increases [41].

Two sequences of terms were used to search for papers using Boolean operators:

(i) Keyword “and” type of building “and” countries “or” geographical words;
(ii) Type of population “and” type of disability “and” area in the house “and” countries “or” geographical words.

Table 1 shows the words and themes used in the search. All the words in Table 1 were used in English, Spanish, and Portuguese.

There were 172 results obtained from documents in each database, classified as follows: 67 results from Scopus, 73 results from Scielo, and 32 results from Latindex. From Google Scholar, more than 213,000 articles were found, for which the first ten pages of results were reviewed. Then, when reviewing the bibliographic references of the 172 articles retained, 21 additional articles were included; most were conference papers with a peer review process.

**Figure 1:**
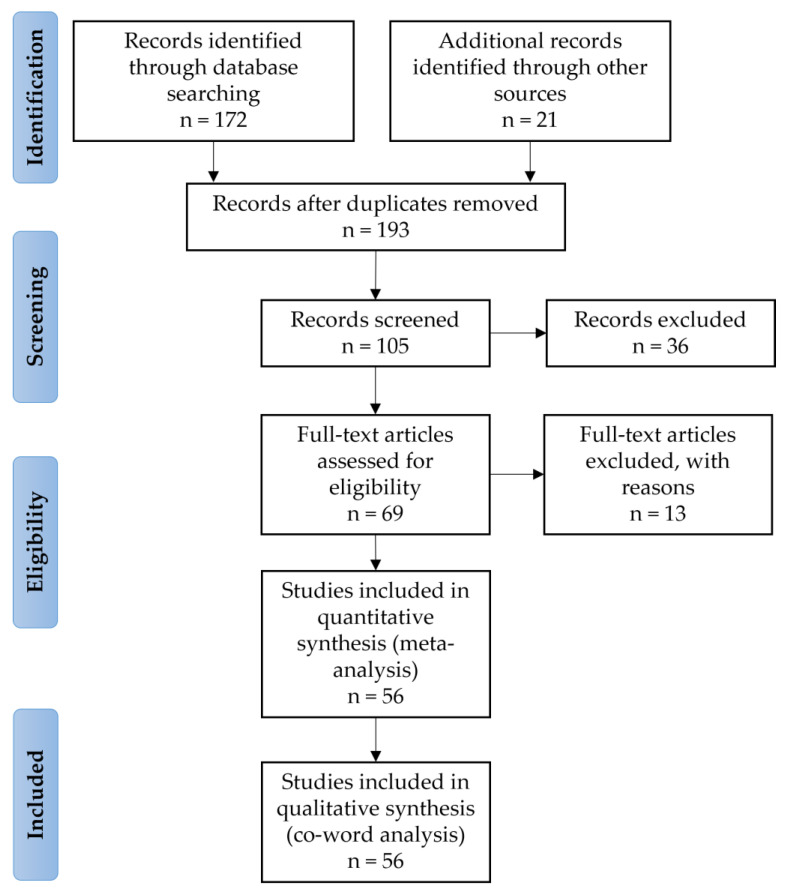
PRISMA Flow diagram of literature search and review. Source: By Authors based on PRISMA 2009 [37].

The authors reviewed the articles, considering the types of studies that contemplated the criteria defined by the researchers. The inclusion criteria were (1) analyses of housing; (2) studies on accessibility; (3) papers developed in Latin America; (4) articles published between 2000 and 2022 (since the UN has recognised the rights of persons with disabilities only since 2006 [42], the range of years was considered from 2000 to cover the entire century); (5) articles written in English, Spanish, or Portuguese; (6) papers published in peer-reviewed journals; or (7) peer-reviewed conference papers. Figure 1 shows the PRISMA flow chart.

The exclusion criteria were applied to 36 articles from the “screening” stage because the discussion was not about accessibility in housing.

After verifying that they were not peer-reviewed conferences, 13 papers were excluded at the “eligibility” stage.

Finally, after the different stages of identification, screening, and eligibility, for the quantitative analysis (meta-analysis) and the qualitative analysis (co-word analysis), 56 articles were included.

### 2.1. Selection and Classification of Articles

For this study, information has been collected from articles related to accessibility in housing, narrowing the spectrum to the Latin American region. The focus is on observing how architectural features, ergonomic interventions, or regulatory issues help improve existing housing for people with disabilities.

### 2.2. Analysis by Types of Variables

After the classification, each of the documents was analysed quantitatively, making graphics to contrast the results and to see if there were any trends or distributions on which countries have developed this type of research, in what area of housing, for what type of accessibility, and in what type of population.

### 2.3. Selection of Articles Based on the Selected Criteria

Finally, based on the criteria selected for analysis, the aim is to understand what aspects and how accessibility is or is not achieved in a dwelling. Table 2 shows the 56 papers retained for the analysis.

### 2.4. Information Analysis Procedure

For the information analysis, the data from the articles were extracted to an Excel spreadsheet to make graphics and tables. Then the titles, keywords, and summaries were processed and exported to VOSviewer software (version 1.6.18) [99,100] for the qualitative analysis, obtaining the concept maps and performing a cluster analysis to identify the main lines of research on accessibility in housing.

The VOSviewer software applies an association strength normalization by default. Then, after selecting the top 60% of recurring terms, a relevance score is calculated, finally showing show the association strength of those terms.

## 3. Results

The primary analyses of the selected papers are divided into four parts: the first part shows the geographical distribution of accessibility studies in Latin America. The second part describes the type of disability for which these analyses are carried out and the type of spaces. The third part presents the analysis of co-words. Finally, in the fourth part, different approaches used to improve the accessibility of housing in LATAM, i.e., through regulations, technology, or architectural or ergonomic interventions, are discussed.

### 3.1. Geographical Distribution and Housing Areas Studied

**Figure 2:**
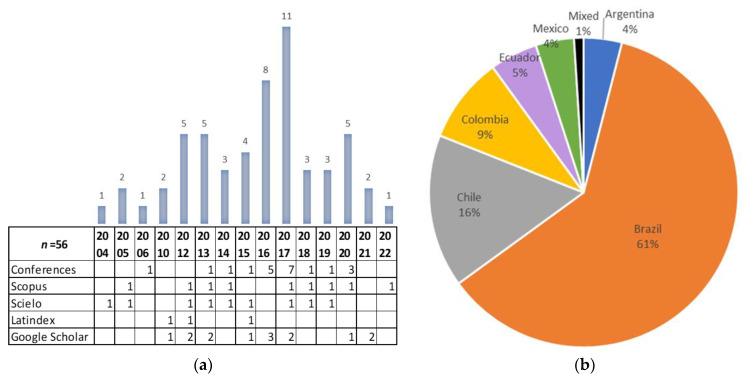
Distribution of selected publications (**a**) by year and by type of database, and (**b**) by countries studied.

As shown in Figure 2a, the research on accessibility starts from 2004 to 2010 with low productivity, even with non-productive years, such as 2007, 2008, and 2009. There is a more or less stable period of 3 to 5 papers between the years 2012 and 2015, reaching its highest productivity in 2017 with 11 papers, thanks mainly to conference papers. In the years 2018 to 2021, it is more or less stable.

On the other hand, in Figure 2b, it is observed that the countries in Latin America that have mainly been researched in terms of accessibility in housing are Brazil at 61%, followed by Chile at 16%, Colombia at 9%, Ecuador at 5%, and then Mexico and Argentina with 4%. Finally, mixed classification represents only 1% of the studies, with three countries—Argentina, Paraguay, and Uruguay—analysed and compared in terms of the regulations on accessibility in those countries [93].

### 3.2. Type of Disability and Type of Population

**Figure 3:**
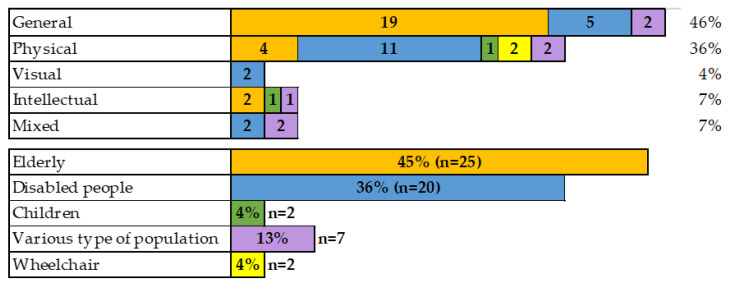
Distribution of the type of disability and type of population studied.

In the distribution by type of accessibility (first graph, Figure 3), the so-called “general” group predominates with 46%, in which no specific disability is analysed, but mainly regulatory issues for disabled people (*n* = 5) [49,54,64,82,93] or housing improvements for disabled people (*n* = 20) [44,45,48,53,55,58,65,67,68,73,76,80,84,85,86,90,91,96,97,98].

In the second place is found the group with a physical disability (36%), with a strong focus on reduced mobility [43,47,57,59,60,61,66,70,72,74,77,78,79,81,83,87,88,89,94,95].

The group of visually impaired people (4%, *n* = 2) are mainly related to technological devices used inside the housing to improve the quality of life of this type of people [56,71].

Under intellectual disability (7%, *n* = 4), products for the daily activities of people with Parkinson’s (P) [75], and requirements for older people with Alzheimer disease [68] are analysed, as well as identification of the needs of autistic people regarding their relationship with the built environment [62,92].

Finally, a mixed group, with 7% (*n* = 4), includes papers in which there were one or more people with different conditions in the housing. In this group, the papers of Cobra and Wataya [50] and Coral et al. [51] stand out, in which they analysed the use of technology in housing for people with reduced mobility and vision problems. This also includes a second group, in which there was a mix of people with a disability situation. For example, Narváez-Tijerina, Fitch Osuna, and Vásquez Rodríguez [69] propose architectural remodelling of housing for the older people or children, and for the physically or visually impaired, while Hechavarría Hernández, Forero, and Vega Jaramillo [63] propose a housing design for a family composed of an older man with reduced mobility and a young man with Down syndrome.

Regarding the types of populations studied in the articles (see second graph, Figure 3), accessibility for the older people is found to a greater extent, with 45% (*n* = 25). This group analyses where the most significant architectural barriers are found inside the dwellings preventing the older people from carrying out their daily activities without problems [43,44,45,48,53,55,58,65,67,68,73,74,75,76,79,80,81,84,85,86,90,91,96,97,98].

For the second most important group, disabled people (36%), as mentioned above, the main issues analysed are housing regulations [47,49,54,59,64,66,82,83,87,93], use of technology [50,51,56,71], and accessibility problems for people with physical disabilities [60,72,78,88,89,94].

In the papers on children (4%, *n* = 2), the adaptation of the bedroom of a child with autism is analysed [62], while Gasporoto and Alpino [61] evaluated the home accessibility of children using wheelchairs or walkers.

For the wheelchair user group (4%, *n* = 2), two authors present architectural criteria to help architects, engineers, and designers design more accessible social housing [57,77].

Finally, in the papers where there are several types of populations, at 13% (*n* = 7), the populations are analysed in parallel: disabled people and older people [46,52,63]; older people and children [69]; disabled people and caregivers [92]; older people and wheelchair users [95]; and older people, caregivers, and wheelchair users [70].

### 3.3. Type of Space and Type of House

**Figure 4:**
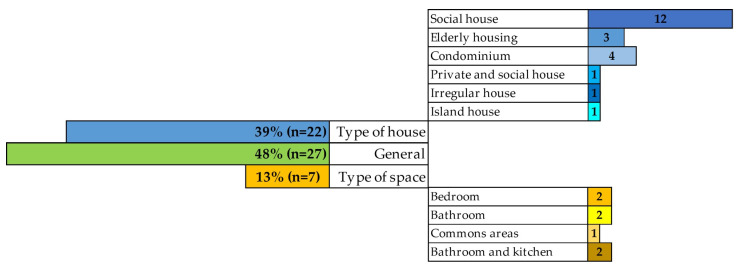
Distribution of the space or type of housing studied.

Regarding the space or type of housing (detailed in Figure 4), most papers are not conducted in a specific housing space or type of housing (48%), or the article does not indicate this information.

Thus, two groups are divided from the other 52%: types of housing analysed (39%) and the type of space in the housing analysed 13%.

In addition, six groups are distinguished by the types of housing analysed: social houses, housing for the older people, condominiums, private and social houses, irregular houses, and island houses. The most prevalent is the analysis of accessibility in social housing, with 12 studies, since, as pointed out by several authors, the accessibility criterion is mandatory for housing developed by the state [46,49,57,63,69,72,77,78,82,87,95,96]. There is even research comparing the regulatory accessibility status of private and social housing [59]. Moreover, there are two groups, each with more than 10%, where the accessibility of housing built for the older people (*n* = 3) is analysed [73,80,84], or accessibility in condominium housing (*n* = 4) [53,70,90,91].

A study of irregular-type housing [88] demonstrated the poor relationship between the criteria defined as accessible and the real needs of users. Moreover, another study, of isolated housing for the older people on an island near a river, showed that, despite general satisfaction on the part of the interviewees, accessibility and safety are the worst evaluated aspects [55].

In the group covering spaces inside dwellings, only 13% of the papers sought to analyse accessibility in a specific area of the dwelling, with the principal places of analysis being the bedroom (*n* = 2) for a bedridden older person [43] and an autistic child [62]. And bathroom (*n* = 2) for a person with dwarfism [89] and another one for older people with Alzheimer's disease [68]. Also, two papers analysed the kitchen and the bathroom [48,94]. Finally, one paper analysed common areas for people with reduced mobility [47].

### 3.4. Co-Word Analysis

A bibliometric design was carried out to understand the main research areas, using knowledge maps, mapping techniques, and word and cluster analysis of the 56 articles selected according to the PRISMA procedure.

This content analysis seeks to generate a pattern of co-occurrence from the words of a sentence in a collection of texts to identify the relationships between ideas in different thematic areas [101].

**Figure 5:**
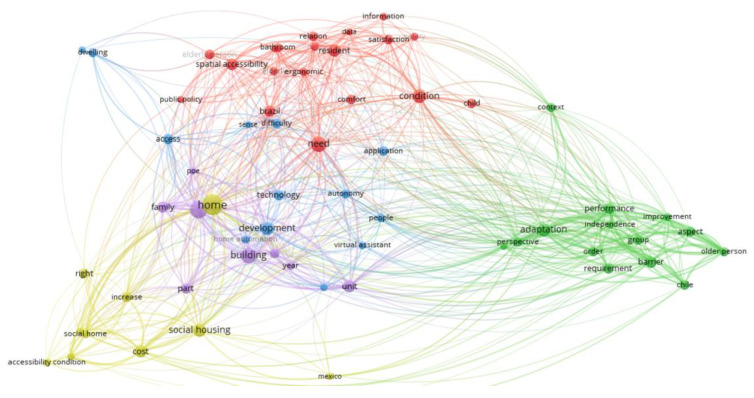
Co-occurrence network of the 56 selected papers.

Thus, the reviewed articles’ titles, keywords, and abstracts constructed a bibliometric network of terms. The diagram in Figure 5 visually represents the relationships between different concepts in a specific field of study (here, accessibility in housing). The nodes represent the most repetitive and, therefore, most researched words, while the size of a node represents the number of word repetitions.

The relationship between the different terms will create a “cluster” observed through the connection network’s “lines” and the different colours of the nodes. In addition, the size of a line gives the strength of the grouping or association of the topic; i.e., two words joined with a short line means that these terms are investigated several times in different investigations. Thus, the results of the diagram allow for a quick visualization. For identification of the most researched groups of topics in a set of scientific articles or an individual research topic, on the other hand, a quality function is used to maximise the grouping of the words in a specific cluster. This function is detailed in the work of VanEck and Waltman [101].

The bibliometric analysis of the 56 retained articles provides 690 networks (lines) between 65 selected terms provided by the program, represented in circles (nodes). The terms are differentiated by colour (red, green, blue, purple, and light green), showing five thematic clusters predominating in the selected papers.

Figure 5 allows us to quickly, and graphically, visualise the less studied or mentioned topics in the literature, such as “dwelling” or “Mexico”. In addition, it allows us to see how these terms are related; i.e., when there are repetitions of a set of words, different clusters are created, marked in colours, and then zooming in on that set of repetition of words allows us to identify significant research themes.

Looking at Figure 5, “home” is the most repeated term in the analysed articles (larger size of light green), unlike “dwelling”, with a small circle represented by a blue node. On the other hand, an intense strength of association is observed between the blue and the red cluster, due to the distance, and proximity of words, unlike the green cluster, which is farther away from the centre.

In the following section, each research area (the clusters) resulting from the biblio-metric analysis will be analysed in more detail. Moreover, the papers that contributed to creating those research areas will be discussed.

## 4. Discussion

By analysing the central nodes of each cluster, the co-word analysis makes it possible to identify the major thematic areas of research. Thus, in the 56 selected articles investigating housing accessibility in LATAM, four sub-areas of research stand out (light green, blue, red, and green clusters): analysis of regulatory issues, technologies, ergonomic interventions, and architecture and welfare.

### 4.1. Analysis of Regulatory Issues

**Figure 6:**
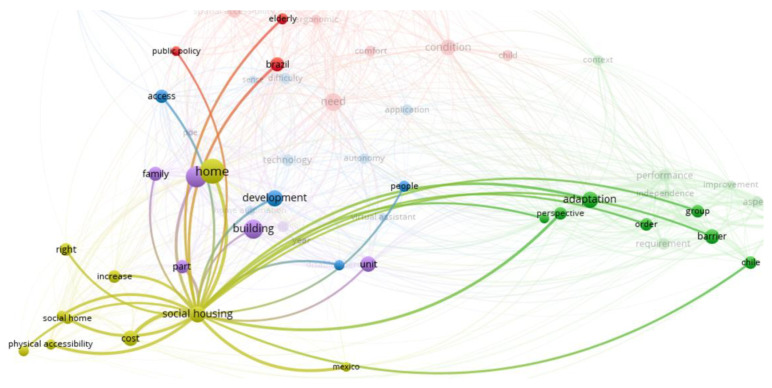
Regulatory issues cluster.

Figure 6 shows that the most searched words in this cluster are home, social housing, and cost. Social housing and cost as significant concerns are closely related, which may seem natural since one of the main concerns when designing and building social housing is always the associated cost, which becomes more relevant in the case of affordable housing.

It is also observed that the words “adaptation” and “barrier” are strongly associated with other clusters since, as explained below, several studies analysed these issues in Chile, Mexico, and Brazil (dark green, light green, and red nodes, respectively).

Thus, this cluster (Figure 6) groups papers describing regulatory developments, regulatory compliance, cost analysis, or technical issues that allow the adaptation of housing for regulatory compliance related to accessibility, with a strong focus on people with physical disabilities and in the social house.

In the group of regulatory issues only, a paper by Barbosa [44] stands out, which analyses public policies highlighting the rights of the older people, mainly in Brazil, identifying that the country is unprepared to receive this growing population. De Souza and Righi [54] do something similar by analysing the evolution of accessibility concepts in Brazil and the importance of accessible spaces. They conclude that although there are standards and laws (considered the most advanced in the world) in Brazil, they still need to be fully and satisfactorily implemented to promote the social inclusion of people with disabilities. In Colombia, Hernandez Posada [64] analysed the regulations for compliance with accessibility, and Morales Mortero [67] analysed the accessibility criteria for housing for the older people. Finally, Vaccotti [93] analyses the right to adequate housing through the description, interpretation, and comparative analysis of how legal norms have evolved in Argentina, Paraguay, and Uruguay from 1990–2010.

Regarding regulatory compliance, two research studies in Brazil analyse regulatory criteria for housing. In research by Caldas, Moreira, and Sposto [47], accessibility conditions in common spaces show that old buildings are the most critical. Research conducted by Calado and Elali [46] also analysed the degree of accessibility in social building, demonstrating that the regulatory criteria are not met. In Colombia, Carrizosa Bermúdez [49] analysed the quality of social housing projects through an evaluation that showed that the scores obtained for architectural attributes in state-subsidised projects are not substantially better than in private housing.

Regarding the technical analysis to achieve regulatory compliance related to accessibility, Santos, Oliveira, and Sposto [82], by analysing architectural projects of social housing and criteria of Brazilian regulations, identify that a significant non-compliance is with the space required to move inside the housing, especially with the use of a wheelchair. Serrano Guzmán, Solarte Vanegas, and Pérez Ruiz [83] present evidence of accessibility problems in different Colombian residential projects related to a lack of compliance with regulations and monitoring during construction, physical barriers to accessibility in bathrooms, bedrooms, and common areas, as well as the management of lighting in housing. Finally, Solis-Carcaño, Utsuki-Alexander, and Vera -Manrique [87] evaluate a group of Mexican housing units built for people with disabilities. The main findings were an unsatisfactory level of compliance with the requirements for accessibility and a perception that the housing was moderately accessible.

Three Chilean studies associated with the analysis of costs to achieve regulatory compliance related to accessibility stand out. Ferrada, Valderrama and Fuentes Contreras [59] carried out a technical study of non-structural modifications to social and private housing to achieve regulatory compliance, showing that, in private housing, this is achieved by increasing space by 0.08 US/sq.m, and, in social housing, by 0.05 US/sq.m. Montoya and Valderrama-Ulloa [66] analysed the degree of accessibility compliance in five private apartments, demonstrating that, with non-structural modifications (e.g., moving partitions), the costs of modifications are almost negligible. Orrigoni et al. [72] analysed the cost adaptation of 15 social housing units to meet accessibility criteria. In 13 of them, the construction cost increased by 1.27% to 6.11%, but the unit cost per square metre remained the same; this is mainly due to having to increase built-up areas. Finally, Narvaez-Tijerina, Fitch Osuna, and Vásquez Rodríguez [69] analyse the investment needed to adapt housing in Mexico, estimating that the average percentage of the cost over the total value of the poorly adapted housing stock in the country is 2.13%.

Thus, this group of papers shows that although some LATAM countries have increased the application and creation of housing accessibility standards, they are not fully complied with. It is necessary to increase monitoring efforts while implementing new projects and to extend their application to existing projects.

### 4.2. Technologies

**Figure 7:**
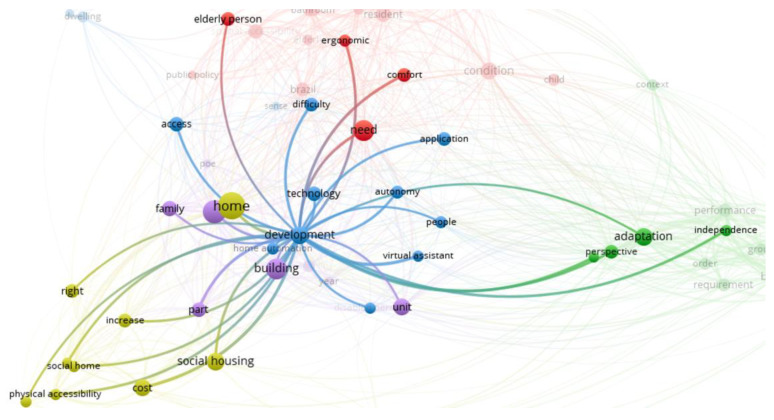
Technologies cluster.

Figure 7 shows that the words that make up this cluster are access, application, technology, difficulty, development, autonomy, and virtual assistant, and that the most researched words in this cluster are development, technology, domotics, and need. Domotics and development are two terms that have a strong relationship since there is currently much interest in incorporating technologies that contribute to improving the quality of life of people, which becomes more relevant when associated with issues of disability, inclusion, and accessibility.

On the other hand, this figure shows that this cluster strongly associates with the neighbouring clusters. It is more difficult to isolate it as a single topic since the development of technologies also has a direct impact on making modifications to housing (the group of words in the dark green cluster), together with the cost analysis that allows these modifications to be made (the group of words in the light green cluster).

Thus, this group includes research that analyses or develops technology to assist in the daily life of people, with a strong focus on older people and visually impaired people.

Abarca et al. [43] analyse the use of technology for bedridden older people. In a study by Dantas Filho et al. [52], a literature review demonstrates the advantages of using technology to increase accessibility. An article by Sobral, Paiva, and Vllarouco [86] identifies the preferences and desires of the older people concerning the physical environment through the Visual Selection technique. At the same time, the research of Pessoa et al. [75] analysed a 3D Assistive Technology product for the daily living activities of patients with Parkinson’s disease.

Cobra and Wataya [50] developed a virtual assistant with voice command to assist people with visual impairment and low mobility to promote greater practicality and functionality in the home environment according to the needs of people with disabilities. In another study, Coral et al. [51] developed a home automation system to solve the problems of accessibility and comfort inside the homes of people with visual and motor disabilities. In addition, dos Santos et al. [56] developed a navigation device inside homes for people with visual disabilities. At the same time, Eyng, Guglielmi, and Borsatto [58] created an architectural manual for older people that helps with autonomy in the performance of daily activities using assistive technologies. Finally, Oliveira et al. [71] researched the perceptions of 20 visually impaired Brazilians on the use of domotics in housing.

This area of research is still incipient, mainly due to the low development of technologies for use by disabled people. However, the boom in virtual assistants, automation systems, and navigation equipment is noteworthy. Therefore, there is an excellent opportunity for development, mainly to improve the autonomy of disabled people in their daily tasks.

### 4.3. Ergonomic Interventions

**Figure 8:**
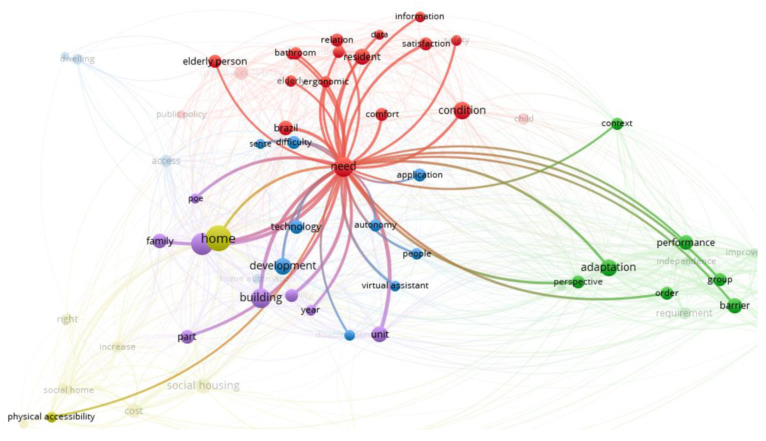
Ergonomic intervention cluster.

Figure 8 shows that the most researched words in this cluster are need, condition, resident, older people, bathroom, ergonomic, comfort, and satisfaction. Two pairs of terms have a strong relationship: need and Brazil and need and comfort. The first is associated with the number of studies Brazil has developed in recent years. It stands out as the Latin American country with the most studies on accessibility issues. In the second case, it is observed that from an ergonomic point of view, it is relevant to have comfortable spaces that respond to people’s needs. In the case of accessibility issues, these needs are often not fully met, so it is important to learn more about them.

This set of words in the papers analyses the use of ergonomics for spatiality and the study of ergonomic interventions to improve accessibility and safety for people with disabilities.

Camara et al. [48] analyse and apply ergonomics criteria in the kitchen and bathrooms of the older people, proposing changes and adaptations of distribution to make kitchens and bathrooms safer and more comfortable for this population. In a study by De Brito et al. [53], spatial accessibility in a multifamily residential condominium for the older people in Brazil is evaluated. The results show that the internal spaces of the apartments, composed of a kitchen and bathroom, are tiny, which may hinder the movement of the older people with restricted mobility (wheelchair, walker, or another mobility prosthesis). In terms of the external areas, the results indicate the lack or poor installation of handrails and the presence of ramps with inadequate slopes.

A study by Martelli and Colle [68] aims to reveal the environmental needs that promote a comfortable, cosy home for the older people with Alzheimer's Disease. Oliveira and Elali [70] analyse the adaptation of environments with small areas in two dwellings against ergonomic criteria. The results show the technical possibility of modifying the units. However, these modifications have a high price (social, economic, and environmental) because the space of the projected units is generally not flexible.

Paiva, Ferrer, and Villarouco [73] apply the Ergonomic Methodology for the Built Environment to study collective residences for the older people, seeking to identify configurations of these dwellings with shortcomings or successful solutions.

Finally, Tavares et al. [89] show that, despite using the Brazilian standard or anthropometric tables, there are many difficulties in designing a bathroom for dwarfs.

In the research on this topic, it was observed that ergonomic intervention sought to solve some of the shortcomings related to the design and the lack of collaborative work in different specialities in order to understand the real needs of people with disabilities when using the housing. The studies also find that housing design often limits functionality and does not meet the needs of different populations, particularly in terms of comfort, accessibility, and safety.

### 4.4. Architecture and Welfare

**Figure 9:**
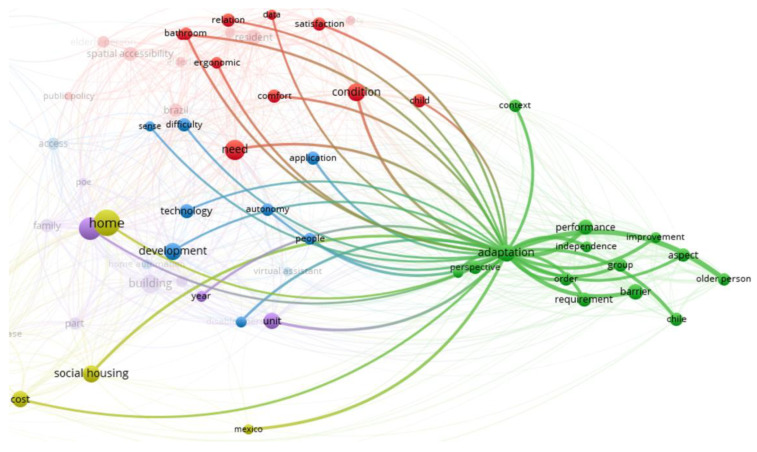
Architecture and welfare cluster.

Figure 9 shows that although this cluster is more isolated from the others, there is an association with the other clusters. The most researched words in this cluster are adaptation, perspective, independence, performance, requirement, improvement, and older person. Adaptation and perspective have a strong relationship since it is understood that modifying or adapting a dwelling is one of the existing possibilities when addressing the issue of accessible housing. The question always arises as to whether it is better to design such a dwelling from the beginning or to modify an existing one only when a family or individual requires it.

Thus, this cluster brings together studies on the analysis of architectural criteria or barriers to independence in the daily life or welfare of people with disabilities.

In architecture, Gonçalves da Silva and da Silva Barros [62] propose improvements to the convenience and safety of autistic children using the User-Centred Construction Project Methodology, which guides the process on the adequacy of children’s bedrooms, focusing on the comfort and welfare of this group. The study by Pereira and Palermo [74] presented a theoretical framework for using living space in a group of users with restrictions. Their main physical, cognitive, and sensory characteristics as well as physical accessibility and accessibility criteria are evaluated and applied to different analysis cases. Pizzi et al. [76] describe and apply an assessment tool designed to detect physical barriers and risks in the performance of basic activities of daily living by older adults, demonstrating that the design of social housing limits functionality in a third operation. The study by Prado et al. [77] provides different design criteria that must be incorporated to make accessible the 15 dwellings studied. Troncoso and Cavalcante [92] seek to draw attention to the needs of autistic people in terms of their relationship with the built environment. Hechavarria Hernández, Forero, and Vega Jaramillo [63] deliver an inclusive design proposal for social housing in Ecuador for a family composed of an older adult with motor disabilities and a young man with Down syndrome.

Regarding the analysis of architectural or spatial criteria, an article by Tabbal, Piccoli, and Quevedo [88] verifies the quality of life perceived by eight people with physical disabilities in demhab affordable housing and evaluates an architectural design project. The average perception of their quality of life was found to be regular. In the environment domain, the home environment item obtained the highest average among all items, and in that same domain, the financial resources item had the lowest average.

Ricaurte Romero and Hechavarría Hernández [78] develop an analysis of a social housing complex in Ecuador for a population with significant physical disabilities, and conditions of vulnerability, marginality, and extreme poverty. The study shows that 35% of the homes surveyed are overcrowded, with only 21% of the families reporting non-conformity. The perception of problems and deficiencies in the construction of the housing was high. Users reported thermal comfort problems because the design did not consider bioclimatic criteria. In addition, there were complaints about deficiencies in the provision of basic services that have repercussions for the healthiness of homes.

Gaete-Reyes, Acevedo, and Carraha [60] proposed a research methodology to study the access and spatial experience of people with disabilities in their homes and neighbourhoods. It is based on combining architectural design methods and audiovisual ethnographies.

The main objective of a study by Gasparoto and Alpino [61] was to evaluate the accessibility of home care for five children with physical disabilities. A lack of furniture or resources adapted to children was identified, despite frequently indicating the need for locomotion, hygiene, and dressing assistance. Among the universal barriers evaluated, the most relevant issues included uneven walkways, upward pathways, and slick walking surfaces on the access routes, and the inadequate door width of the bathroom. 

Romero et al. [79] analysed 411 interviewees regarding the spatiality of the residents’ dwellings, where low comfort and functionality for the young and the older people were detected.

Salcedo, Magagnim, and Pereira [80] analysed the quality of the built environment in social housing for older adults, showing that dwellings with kitchenettes and one-bedroom apartments are insufficient to meet the needs of the older people due to the lack of space for the performance of daily activities.

Silva et al. [84] verify aspects of accessibility in a social housing project for 30 dwellings in Brazil. The absence of technical criteria and equipment that support accessibility was identified in the building and urban environments, producing difficulties in accessing other spaces, and mobility difficulties, both in the central environment and in the environment.

Yeannes [94] describes adapted designs for the kitchen and bathroom, highlighting the importance of including the disabled person in the modifications and even re-educating them in their habits.

Finally, in the Yoshida group of studies, residents’ satisfaction with different projects concerning accessibility is analysed. Thus, in a study by Yoshida and Magagnin [97], seven older people in Brazil were interviewed about issues relating to ease of movement and the arrangement of furniture within each environment; ease in the use of furniture and equipment; the level of lighting and ventilation in environments; thermal comfort; and safety when moving between environments. In another study by Yoshida and Magagnin [98], the degree of satisfaction of 22 older people was evaluated, indicating problems with the type of floor, ease of opening and closing of a door (type of handle), ease of opening and closing of a window, space to use the shower, ventilation problems and natural lighting, and area available for circulation. In a study by Yoshida et al. [95], they demonstrate that, although the studied social residences of older people were designed according to universal design principles, the environments do not allow for the linear movement of a wheelchair user. Furthermore, Yoshida and Magagnin [96], when evaluating the degree of accessibility for a wheelchair in an apartment in the 1990s in Brazil, showed that although the country had a technical standard of accessibility during the period of construction of the building, these principles were not included in the project, because some environments did not allow for the free movement of the wheels of the user’s wheelchair, especially given the reduced space of the bathroom, kitchen or service area, and the area available for circulation.

In this first group, the studies highlight the importance of user-centred design, considering the user’s physical, cognitive, sensory, and evolutionary (specific to children) characteristics and needs. On the other hand, the analysis of architectural criteria sought to reduce physical barriers and risks for people with disabilities. Finally, a group of studies provided criteria, proposals, and architectural models to design or create accessible housing.

The analysis of welfare and daily living is highlighted in a study by Briede-Westermeyer and Pérez-Villalobos [45], who interviewed 35 older people in Chile with the strategy ‘A day in the life of …’. The results show that the routine of the older adult is characterised by a diversity of tasks and purposes and a usually positive view of everyday life.

Lujan [65] interviewed 100 older people to evaluate fall risks in housing and public spaces. In the case of housing, behavioural factors are the main reason for falling.

Do Nascimiento et al. [55] investigate the housing conditions of 23 older adults living near the Amazon River and evaluate their satisfaction with their housing. Despite high territorial isolation, low socioeconomic status, and largely inadequate housing conditions, the results reveal the overall satisfaction of older adults with their home environment, except in terms of accessibility and safety.

Teston and Marcon [91] compare the quality of life of 50 older people living in a geriatric institution with 173 older people living in the community. The groups differed significantly in physical appearance, environment, sensory functioning, and participation, which was better rated in the geriatric institution, while community-dwelling residents scored higher in the intimacy domain.

Teston and Marcon [90] develop a study to learn how 20 residents of an “older people condominium” perceive the quality and conditions of their life in a new housing modality. The factors valued by the older people in their perception of quality of life were independence, autonomy, having an occupation, developing leisure activities, acceptance of the ageing process, as well as the type of structure and characteristics of the housing modality, due to the possibility of social interaction.

Finally, this second subgroup highlights aspects of the relationship people with disabilities have with their housing in their daily lives, routines, and needs. These sociological investigations allow us to visualise factors related to well-being—that are not necessarily found in the physical aspects of housing but in the environment (comfort)—as well as the importance of privacy and social interactions.

## 5. Conclusions

This article presents a systematic literature review in order to understand the current state of research on accessibility in housing for people with disabilities in Latin America. To develop this study, the PRISMA methodology was used, retaining 56 papers for analysis. After that, co-word analysis was used to identify common themes and topics within the articles. The results allow for answering the research questions. First, regarding the countries in Latin America that have researched accessibility in housing, it was found that Brazil, Chile, Colombia, Ecuador, Mexico, and Argentina are the ones with the most significant number of studies. Second, regarding the type of disability studied, the so-called “general” group predominates, in which no specific disability is analysed, but mainly regulatory issues for disabled people or housing improvements for the older people in line with the demographic changes experienced by countries in Latin America, followed by physical disability. The analysis of the different types of disability and types of population suggests that it is necessary to include other types of disabilities, such as visual or cognitive, as well as other types of the population, such as children, young adults, and caregivers.

In addition, the study showed that housing accessibility in LATAM presents some barriers that must be addressed. First, there is an insufficient supply of housing that meets accessibility standards and a lack of access to the means to acquire or rent accessible housing. This may be due to financial and socioeconomic limitations, slow implementation of updated regulations, or lack of information and data.

Regarding the co-word analysis presented in the results section, four major thematic areas of research in LATAM have been highlighted: regulations, technology, ergonomic interventions, and the architectural and well-being aspects of housing, with the following conclusions:

- Although there is an increase in the development of accessibility regulations in housing, it has not been fully implemented, and it is necessary to improve its supervision during the implementation of projects and extend its compliance to existing housing.
- In terms of technology, although its development in LATAM is incipient, it promises a great field of expansion in support of people with disabilities in the functionality of the home environment and in the performance of their daily activities.
- Regarding housing design, ergonomic interventions become necessary mainly because the architectural design limits functionality and does not meet the needs of the different populations present in the home, especially in terms of comfort, accessibility, and safety.
- In the architectural aspects, the main objective is to reduce physical barriers and risks for disabled people or to provide criteria, proposals, or architectural models to design and/or create accessible housing. Finally, regarding the welfare of people with disabilities and their daily life in their homes, thermal comfort, the importance of privacy, and social interactions in their daily lives are emphasised.
- Finally, as these results are part of a broader investigation into housing accessibility issues, future work will continue these topics. The research team is developing a broader architectural standard to inform users more clearly whether or not a home is accessible. A multi-criteria evaluation method for the accessibility level of new housing will be created to complement these standards so that people can make informed purchases. In addition, based on the knowledge acquired regarding problems, barriers, and other issues regarding housing accessibility, it is expected to develop public policies for the existing housing stock and to improve subsidies. Finally, a broader multidisciplinary framework will be applied to reach the best results in this research, as accessibility is a multivariable issue.

## Figures and Tables

**Table 1:** Keywords and themes used in the search.

Keywords	Type of Building	Type of Population	Type of Disability	Area in the House	Countries	Geographical Words
Inclusive	Housing	Children	Physical	Bedroom	Argentina	Developing countries
Accessibility	House	Older person	Visual	Bathroom	Bolivia	Low-income countries
Disability	Home	Caregivers	Hearing	Kitchen	Brazil	Middle-income countries
Impairment	Dwelling	Parents	Intellectual/cognitive	Living room	Chile	Hispanic world
	Apartments	Disabled people		Dining room	Colombia	Central America
	Residential building	Wheelchair user		Internal life space	Ecuador	Latin America or LATAM
	Condominium				Paraguay	South America
	Social house				Peru	
	Private house				Uruguay	
					Venezuela	
					Mexico	

**Table 2:** Documents included in the systematic review (n = 56). Source: by authors.

Author	Year	Country	Type of			
			House	Space	Population	Disability
Abarca et al. [43]	2018	Chile	general	bedroom	older people	physical
Barbosa, Ferreira [44]	2021	Brazil	general	general	older people	general
Briede-Westermeyer, Pérez–Villalobos [45]	2019	Chile	general	general	older people	general
Calado, Elali [46]	2016	Brazil	social housing	general	disabled people, older people	general
Caldas, Moreira, Sposto [47]	2015	Brazil	general	common areas	disabled people	physical
Camara, Engler, De Oliveira Fonseca [48]	2010	Brazil	general	kitchen, bathroom	older people	general
Carrizosa Bermúdez [49]	2010	Colombia	social housing	general	disabled people	general
Cobra, Wataya [50]	2020	Brazil	general	general	disabled people	visual/physical
Coral et al. [51]	2019	Colombia	general	general	disabled people	visual/physical
Dantas Filho et al. [52]	2021	Brazil	general	general	disabled people, older people	general
De Brito Prado et al. [53]	2015	Brazil	condominium	general	older people	general
De Souza, Righi [54]	2016	Brazil	general	general	disabled people	general
do Nascimento et al. [55]	2017	Brazil	island house	general	older people	general
dos Santos Roque et al. [56]	2017	Brazil	general	general	disabled people	visual
Dos Santos, Santos, Ribas [57]	2005	Brazil	social housing	general	wheelchair user	physical
Eyng, Guglielmi, Borsatto [58]	2020	Brazil	general	general	older people	general
Ferrada, Valderrama, Fuentes-Contreras [59]	2020	Chile	social and private housing	general	disabled people	physical
Gaete-Reyes, Acevedo, Carraha [60]	2019	Chile	general	general	disabled people	physical
Gasparoto, Alpino [61]	2012	Brazil	general	general	children	physical
Gonçalves da Silva, da Silva Barros [62]	2017	Brazil	general	bedroom	children	intellectual
Hechavarría Hernández, Forero, Vega Jaramillo [63]	2020	Ecuador	social house	general	disabled people, older people	physical/intellectual
Hernández Posada [64]	2004	Colombia	general	general	disabled people	general
Lujan [65]	2006	Argentina	general	general	older people	general
Montoya-Pareja, Valderrama-Ulloa [66]	2016	Chile	general	general	disabled people	physical
Morales Montero [67]	2013	Colombia	general	general	older people	general
Martinelli, Colle [68]	2017	Brazil	general	bathroom	older people	intellectual
Narváez-Tijerina, Fitch Osuna, Vásquez Rodríguez [69]	2018	México	social house	general	children, older people	physical visual
Oliveira, Elali [70]	2012	Brazil	condominium	general	older people/wheelchair user, caregiver	physical
Oliveira et al. [71]	2022	Brazil	general	general	disabled people	visual
Orrigoni et al. [72]	2012	Chile	social housing	general	disabled people	physical
Paiva, Ferrer, Villarouco [73]	2014	Brazil	older people housing	general	older people	general
Pereira and Palermo [74]	2013	Brazil	general	general	older people	physical
Pessoa et al. [75]	2017	Brazil	general	general	older people	intellectual
Pizzi et al. [76]	2013	Chile	general	general	older people	general
Prado et al. [77]	2012	Chile	social housing	general	wheelchair user	physical
Ricaurte Romero, Hechavarría Hernández [78]	2017	Ecuador	social housing	general	disabled people	physical
Romero et al. [79]	2017	Ecuador	general	general	older people	physical
Salcedo, Magagnin, Pereira [80]	2016	Brazil	older people housing	general	older people	general
Salgado Gracia, Olivera Pueyo [81]	2005	Chile	general	general	older people	physical
Santos, Oliveira, Sposto [82]	2016	Brazil	social housing	general	disabled people	general
Serrano Guzmán, Solarte Vanegas, Pérez Ruiz [83]	2013	Colombia	general	general	disabled people	physical
Silva et al. [84]	2015	Brazil	older people housing	general	older people	general
Silveira, Ely, Vergara [85]	2020	Brazil	general	general	older people	general
Sobral, Paiva, Vllarouco [86]	2017	Brazil	general	general	older people	general
Solis-Carcaño; Utsuki–Alexander, Vera–Manrique [87]	2018	Mexico	social housing	general	disabled people	physical
Tabbal, Piccoli, de Quevedo [88]	2014	Brazil	irregular housing	general	disabled people	physical
Tavares et al. [89]	2016	Brazil	general	bathroom	disabled people	physical
Teston, Marcon [90]	2014	Brazil	condominium	general	older people	general
Teston, Marcon [91]	2015	Brazil	condominium	general	older people	general
Troncoso, Cavalcante [92]	2017	Brazil	general	general	disabled people/caregivers	intellectual
Vaccotti [93]	2012	Mixed	general	general	disabled people	general
Yeannes [94]	2013	Argentina	general	bathroom, kitchen	disabled people	physical
Yoshida et al. [95]	2016	Brazil	social housing	general	older people, wheelchair user	physical
Yoshida, Magagnin [96]	2016	Brazil	social housing	general	older people	general
Yoshida, Magagnin [97]	2017	Brazil	general	general	older people	general
Yoshida, Magagnin [98]	2017	Brazil	general	general	older people	general

## Data Availability

Not applicable.
